# Prosopagnosia is highly comorbid in individuals with probable developmental coordination disorder

**DOI:** 10.1177/17470218241275977

**Published:** 2024-08-13

**Authors:** Katherine Jane Maw, Geoffrey Beattie, Edwin Burns

**Affiliations:** 1 Edge Hill University, Ormskirk, UK; 2 Swansea University, Swansea, UK

**Keywords:** developmental coordination disorder, DCD, dyspraxia, motor, prosopagnosia, face recognition, face perception

## Abstract

Developmental co-ordination disorder (DCD) is characterised by difficulties in motor control and coordination from early childhood. While problems processing facial identity are often associated with neurodevelopmental conditions, such issues have never been directly tested in adults with DCD. We tested this possibility through a range of tasks and assessed the prevalence of developmental prosopagnosia (i.e. lifelong difficulties with faces), in a group comprising individuals who self-reported a diagnosis of, or suspected that they had, DCD. Strikingly, we found 56% of this probable DCD group met recently recommended criteria for a diagnosis of prosopagnosia, with 22% acquiring a diagnosis using traditional cognitive task-based methods. Moreover, their problems with faces were apparent on both unfamiliar and familiar face memory tests, as well as on a facial perception task (i.e. could they tell faces apart). Positive correlations were found between self-report measures assessing movement and coordination problems, and objective difficulties on experimental face identity processing tasks, suggesting widespread neurocognitive disruption in DCD. Importantly, some issues in identity processing in our probable DCD group remained even after excluding participants with comorbid conditions traditionally associated with difficulties in face recognition, that is, autism and dyslexia. We recommend that any diagnostic test for DCD should include an assessment for prosopagnosia. Given the high prevalence of prosopagnosia in our probable DCD group, and the positive correlations between DCD and prosopagnosia symptoms, there may be a stronger link between movement and facial identity abilities than previously thought.

## Introduction

Developmental coordination disorder (DCD)/dyspraxia is one of the most common neurodevelopmental conditions (Richardson & Ross, 2000), comprising 6% of the world’s population (Jasmin et al, 2018), yet it remains significantly under-researched with many questions still to be answered (Biotteau, 2016; Cairney et al., 2013; Meachon, 2023; Meachon et al., 2022). DCD affects movement and coordination skills throughout the lifespan (Maw et al., 2024; Purcell et al., 2015; Williams et al., 2017; Zwicker et al., 2018) although intelligence appears to be unaffected (Izadi-Najafabadi et al., 2019; Omer et al., 2019; Sumner, Pratt & Hill, 2016). The condition often leads to delayed milestones in motor activity such as rolling, crawling, walking and running (Sumner, Leonard, & Hill, 2016). It also severely interferes with daily life and self-care activities (O’Dea et al., 2020; Rinat et al., 2020) such as dressing (Jasmin et al., 2018), brushing one’s hair (Missiuna et al., 2007), doing up buttons and zips (Kirby et al., 2010), applying make-up (Cleaton et al., 2021), taking part in physical activities including sports (O’Dea et al., 2020), driving (Cleaton et al., 2021; Kirby et al., 2010) and handwriting (Missiuna et al., 2007). Movements, which others take for granted, are often challenging and upsetting for those with DCD (Harris et al., 2021).

The DSM-5 recommends that to diagnose DCD a person must exhibit the following traits: (a) The acquisition and execution of coordinated motor skills is substantially below that expected given the individual’s chronological age and opportunity for skill learning and use. Difficulties are manifested as clumsiness (e.g. dropping or bumping into objects) as well as slowness and inaccuracy of performance of motor skills (e.g. catching an object, using scissors or cutlery, handwriting, riding a bike, or participating in sports). (b) That these motor skills deficits significantly and persistently interfere with activities of daily living appropriate to chronological age (e.g. self-care and self-maintenance), impacting academic/school productivity, prevocational and vocational activities, leisure and play. (c) Onset of symptoms is in the early developmental period and D) The motor skills deficits are not better explained by intellectual disability (intellectual developmental disorder), visual impairment nor attributable to a neurological condition affecting movement (e.g. cerebral palsy, muscular dystrophy, degenerative disorder; American Psychiatric Association, 2013). The International Classification of Diseases 11th Revision (ICD: 2018) also follows the DSM-5 definition and diagnostic criteria, categorising DCD as a Specific Developmental Disorder of Motor Function (Code 6A04; World Health Organization, 2023). In summary, DCD is characterised and diagnosed by lifelong problems with movement and coordination that significantly disrupt daily life.

Researchers point to social perception as another area which may pose difficulties for people with DCD (Cummins et al., 2005; Sumner, Pratt, & Hill, 2016). For example, in childhood DCD is associated with a range of difficulties in interpersonal communication and related skills, including the recognition of facial identity (Sumner, Pratt, & Hill, 2016) and the identification of emotion (Sumner et al., 2018). Sumner, Pratt, & Hill, (2016) tested children using the short-form standardised Benton Facial Recognition Test (BFRT; Benton, 1994) to determine whether children with DCD were able to identify previously unknown faces. In this task, participants must match a target face to its corresponding image within a group containing both the target and novel lures. Sumner, Pratt, & Hill, (2016) found that children with DCD exhibited problems recognising novel faces in comparison to a control sample with a reduction in performance comparable to that found in children with autism. This suggests that those with DCD exhibit problems with facial identity, at least in childhood.

However, Duchaine and Nakayama (2004) have critiqued the Benton test, suggesting that it is possible for people with severely impaired face discrimination abilities to perform within the range of those with intact face recognition skills. Participants may use a feature matching strategy (e.g. eyebrows and hairline matching) due to the simultaneous presentation of the target face and test faces. Thus, the test may not truly measure identity recognition of unfamiliar faces and researchers advise caution in using it on its own when assessing identity recognition abilities (Duchaine & Nakayama, 2004; although see Murray et al., 2022). Instead, it is generally recommended that when measuring facial identity abilities the prosopagnosia index questionnaire (PI20; Shah et al., 2015) should be used, in combination with neuropsychological assessments of different stages of identity processing (Burns et al., 2023), for example, face perception (e.g. Cambridge Face Perception Test; Duchaine et al., 2007), plus unfamiliar (e.g. Cambridge Face Memory Test; Duchaine & Nakayama, 2006) and familiar (e.g. Famous Faces Test; Duchaine & Nakayama, 2005) face memory tests.

Whilst Sumner, Pratt, & Hill, (2016) found facial identity recognition difficulties in children with DCD, no further work has established whether this continues into adulthood. Similarly, it is unclear if a disproportionally high number of those with DCD have problems with faces that could be considered disruptive to everyday life. This is possible given that DCD is commonly comorbid with other neurodevelopmental conditions such as autism spectrum disorder (ASD; Fry et al., 2022; Kilroy et al., 2022; Miller et al., 2021). Motor difficulties are also a clinically significant problem in people with ASD (Miller et al., 2021), and ASD has also been related to facial identity difficulties (Minio-Paluello et al., 2020; Sumner, Pratt, & Hill, 2016), similar to those found in people with developmental prosopagnosia (DP; Bate et al., 2014; Burns et al., 2014, 2017a, 2017b; Burns et al., 2023; Wilcockson et al., 2020). This latter neurodevelopmental condition is characterised by severe difficulties recognising faces throughout the lifespan in the absence of difficulties in vision, lower IQ or brain injury (Biotti et al., 2019; Bobak et al., 2017; Burns & Bukach, 2021; 2022; Cook & Biotti, 2016; Lowes, 2022). People with DP do experience everyday problems (Biotti et al., 2019) as they will often struggle to recognise faces of very familiar people such as close relatives, friends and work colleagues (Bobak et al., 2016, 1976). Given that many neurodevelopmental conditions are associated with elevated levels of prosopagnosia (Cook & Biotti, 2016; Keogh et al., 2021; Sigurdardottir et al., 2018; Sigurdardottir et al., 2015; Svart & Starrfelt, 2022), it seems quite possible that those with DCD will also have a greater prevalence of DP.

Whilst no research has shown directly that developmental prosopagnosia may be present in those with DCD, there are indications in the prosopagnosia literature that the two conditions may be linked (Cook & Biotti, 2016). For example, there are many case reports of motor difficulties being present in developmental prosopagnosia (De Haan & Campbell, 1991; Duchaine, 2000; Kracke, 1994; Kress & Daum, 2003; McConachie, 1976; Svart & Starrfelt, 2022). Moreover, movement-related brain regions may also be involved in face recognition. Specific areas found to be used in both movement and face identity processing include the posterior superior temporal sulcus (de Gelder et al., 2003; Julie, 2012; Reynolds et al., 2015; Sakurai et al., 2016), the dorsolateral prefrontal cortex (Dinkelacker et al., 2011; Gaymard et al., 2017), the fusiform gyrus (Burns et al., 2019; Schwarzlose et al., 2005) and the cerebellum (Dinkelacker et al., 2011; Gaymard et al., 2017; Nicolson & Fawcett, 2011). There is additional evidence that both movement and face identity processing difficulties may be underpinned by lower neural activity between several brain regions such as the primary motor cortex (M1), the caudate, putamen and globus pallidus (McLeod et al., 2014; Rees et al., 2012; Sugiura et al., 2000; Uono et al., 2017). Alternatively, face recognition and movement-related brain areas may be functionally distinct, but rely upon shared brain connectivity tracts that have degraded functioning (Gaymard et al., 2017).

These findings suggest that there may be a commonality between DCD and DP, with difficulties in social perception and movement likely to overlap. However, the prevalence of prosopagnosia in adults with DCD remains unknown, nor is it clear if their problems with facial identity processing are restricted to face memory, or if they are also apparent at the earlier perceptual stage, that is, where they extract the face’s unique identity-related information prior to encoding in memory (Bruce & Young, 1986).

We therefore investigated whether people who self-reported a diagnosis of DCD, or who suspected that they had DCD, also experience face identity processing difficulties such as those found in people with prosopagnosia, specifically addressing the following issues. Firstly, do they as a group exhibit the general symptoms of prosopagnosia, for example, levels of difficulties when recognising faces in everyday life that would warrant a diagnosis? Secondly, can people with DCD discriminate between the subtle differences that make each face unique, that is, do they have problems perceiving facial identity? And finally, do they demonstrate memory difficulties for unfamiliar and familiar faces? Given that identity processing problems are apparent in other neurodevelopmental conditions (Griffin et al., 2021; Sigurdardottir et al., 2015, 2018, 2019), we hypothesised that adults with DCD would exhibit problems in face identity processing. Such a finding would be important because it would show the challenges facing those with DCD in adulthood are not restricted to movement but may also extend to recognising faces. This could compound, or partly explain, the social difficulties and isolation reported by some with DCD (Cairney et al., 2013; Gentle et al., 2020; Jarus et al., 2011; Kilroy et al., 2022; Li et al., 2019). If confirmed, then clinicians should be advised of potential treatments and coping mechanisms for prosopagnosia when providing support for those with DCD.

## Methods

### Participants

To compute the required sample size for the various groups, the Benton face recognition task means and standard deviations from Sumner, Pratt, & Hill, (2016) were employed, generating a Cohen’s *d* of 1.14. Entering this value into G*Power indicated that 14 participants were required in the control and self-reported DCD groups to detect similar effects in our study with 80% power and alpha set to .05. However, the study recruited many more participants than this (32 DCD, 37 Controls), so should be able to detect roughly medium to large effect sizes (i.e. Cohen’s *d* > .71).

Forty control participants were initially recruited through Edge Hill University and social media to roughly match the ages and gender of the probable DCD (pDCD) group. They reported that they did not have DCD, traumatic head injury, hemiplegia or cerebral palsy. These included 12 men, 27 women and one person identifying as non-binary with an age range of 18 to 73 (*M* = 34.7 years). Three control participants reported a diagnosis of ASD (autism spectrum disorder) and thus were excluded. But note, we did not formally assess autism to validate these self-reports as this would have made testing overly onerous for participants (Hoerger, 2010) and was also likely to result in increased dropout rates (Hoerger, 2010; Short et al., 2022). Controls were asked if they had trouble recognising faces in everyday life, that is, did they feel they had prosopagnosia? One 20-year-old female control participant disclosed that while she could recognise people who were personally familiar, she did have significant problems in recognising celebrities. All her test scores except the Famous Faces Test were within normal range so she was included in the control group. Another two control participants reported general troubles with faces. However, these two participants were included in our control group as their prosopagnosia symptom scores were within the normal range on the PI20 (i.e. <60), and we would expect some variation in face recognition ability within the typical population (Astle & Fletcher-Watson, 2020).^1^ We provide further analyses, which did not change our main findings (i.e. pDCD group impaired in face identity processing) with these two controls excluded for completeness (see Supplemental Information). Our final control group for analysis included 37 participants.

Our final sample of self-reported DCD participants comprised 32 adults (male *n* = 9, female *n* = 20, non-binary *n* = 3) ranging between 18 and 73 years (Mean = 35.8). Participants with DCD were recruited via adverts and email campaigns in our local university and through social media requests in online DCD support groups. We initially recruited a sample of 35 potential participants with DCD: 26 who reported having a confirmed diagnosis from a professional. As in previous studies (Harris et al., 2021; Meachon et al., 2022; Tal-Saban et al., 2012), we did not request proof of diagnosis. One reason for this was data protection (Meachon et al., 2022), and this is discussed further in our limitations section. The remaining nine reported that they suspected that they had DCD. Previous studies suggest that many adults who self-report with DCD (Harris et al., 2021; Tal-Saban et al., 2012) have never been able to acquire a diagnosis for a variety of reasons (e.g. reported cost, parental concern about stigma, lack of awareness in schools, and difficulties in finding appropriate public services).

We excluded three of the initial 35 people who reported DCD for the following reasons: first, a 21-year-old male who did not recognise a single famous person, and when we checked familiarity (e.g. ‘Do you know who Donald Trump is?’) he reported that he did not know any of the famous people semantically. As it is extremely unusual to not be familiar with any of our highly famous people (e.g. Barack Obama, The Queen), we thought it reasonable to exclude this participant. Two of the nine people with suspected DCD (39-year-old male and 45-year-old male) did not score below the cut-off of 17 for movement problems in the childhood portion of the Adult Developmental Coordination Disorder/Dyspraxia Checklist (Kirby et al., 2010; MacCullagh et al., 2017), which assesses symptoms of DCD. This is an important consideration as childhood difficulties are required for an official diagnosis of DCD (Diagnostic and Statistical Manual of Mental Disorders, DSM-5; American Psychiatric Association, 2013); as opposed to motor problems which develop or are acquired in later life, perhaps through illness or injury. They were therefore excluded, and 32 pDCD participants were included for analysis. To allay concerns regarding the exclusion of these two participants on our results, we reran our analyses with them included and found similar results, that is, people with pDCD were generally impaired in face identity processing (see Supplemental Information). One 32-year-old female participant did not move any faces on the Cambridge Face Perception Test (CFPT); however, they completed all other tests fully. She was therefore included in all analyses, except those relating to the CFPT.

All participants reported no history of a serious head injury, nor other conditions that could explain their movement difficulties, that is, cerebral palsy, hemiplegia, or muscular dystrophy. One pDCD participant initially reported a head injury but emailed to confirm that this was a very mild concussion that had occurred before the age of 10, so they were included. People with comorbid neurodevelopmental differences such as ASD, ADHD and dyslexia were included in the DCD group because comorbidity is so commonly reported across these conditions (Astle & Fletcher-Watson, 2020; Brimo et al., 2021; Dewey, 2018). For example, children with DCD have between 30% and 50% chance of having ADHD and 14% and 50% chance of having ASD (You et al., 2023). Hence, exclusion based on comorbid diagnoses of this kind would not produce a representative sample of people with DCD (Astle & Fletcher-Watson, 2020). Our analyses were, however, rerun on the pDCD participants who reported no comorbidities that are known to be associated with face identity processing impairments (i.e. autism and dyslexia; Brady et al., 2020; Cygan et al., 2018; Gabay et al., 2017; Krebs et al., 2011; Sigurdardottir et al., 2018). We report these in our Results section.

There were no significant differences in ages (*t*[67] = .02, *p* = .99, Cohen’s *d* < 0.01) or birth sex (*X*^2^[1] = .01, *p* = .92) between the pDCD and control groups. No participants reported any problems with intellectual functioning. The DCD group reported significantly higher levels of education than controls (*X*^2^[1] = 8.66, *p* = .003), indicating that any face identity processing impairments detected in the DCD group were unlikely to be due to intellectual difficulties. Please note, this difference between groups is not expected to affect testing however, as intelligence is not generally linked with identity processing ability (Connolly et al., 2019; Wang et al., 2012). Moreover, our DCD group (*M* = 70.32, *SD* = 13.31) did not exhibit any impairments on the inverted portion of the Cambridge Face Perception Test (*M* = 69.46, *SD* = 17.69, *t*[66] = 0.22, *p* = .82, Cohen’s *d* = 0.05). This implies that low level vision or attentional problems did not account for any face identity processing impairments in our pDCD group either. However, to counter the possibility that education levels were influencing the DCD vs. control group differences in our Results, we ran exploratory regressions using each face identity processing measure as a dependent variable, and Education (not university educated = 0, university educated = 1), Group (DCD vs. Controls), Age and Sex at Birth (Male = 0, Female = 1) as predictors. Every model was significant (all *ps* < .013), with Group a significant predictor (i.e. DCD participants were poorer with faces than controls; all *ps* < .004), except for the CFPT inverted errors (*p* = .56). This suggests people with pDCD exhibit problems with faces even after education levels are considered.

### Materials

#### DCD measures

Due to Covid-19 restrictions, all data was collected online with no in-person motor ability testing; however, it is noteworthy that no specific, agreed movement-related diagnostic tasks currently exist to diagnose DCD in adults (Meachon et al., 2022; Purcell et al., 2015). Moreover, previous studies have shown self-report measures such as the ADC accurately separate DCD and control groups (Cleaton et al., 2021; Engel-Yeger, 2020). As mentioned, we used the AACQ and ADC questionnaires to assess motor abilities. The AACQ includes 12 coordination problem statements based on the diagnostic criteria of the Diagnostic and Statistical Manual of Mental Disorders (DSM-IV-TR; American Psychiatric Association, 2013). The AACQ has been shown to be a valid and reliable tool for assessing DCD symptomatology in adults (Tal-Saban et al., 2012). It requires self-report responses to questions such as, ‘I have difficulty using instruments like: a can opener, a corkscrew, an electric saw or drill, vacuum cleaner, DVD player’, ‘I have difficulty orientating in space like: getting lost easily, difficulty learning how to get to a new place, difficulty recognising familiar driving routes, problems using a road map, difficulty finding your car in a car park, difficulty exiting’ and ‘ I have difficulties with daily activities like: buttoning, lacing shoes, preparing a light meal, cutting nails, shaving, lighting the stove with a match, applying cosmetics, ironing, loading the dishwasher, putting on earrings, closing a necklace tying a tie’. Responses were selected from a 5-point Likert scale ranging from ‘Never’ to ‘Always’. Scores ranged from 12 to 60 with a cut-off for symptomology consistent with having DCD (>26.71; Tal-Saban et al., 2012).

In addition, the ADC contains 40 self-report questions and assesses three areas of common difficulties: (a) previous childhood problems; for example, as a child ‘Did you have problems writing neatly? (so others could read it)’ and ‘Did you have difficulty eating without getting dirty?’ Section (b) motor difficulties; for example, do you currently ‘have difficulty with hobbies that require good coordination?’ and ‘copying things down without making mistakes?’ Finally, part (c) examines executive functioning issues, such as, currently ‘Would you say you bump into things, spill and break things?’ and ‘Do you have difficulty folding clothes to put them away neatly?’ Each question is allocated a score as follows: Never = 0 Sometimes = 1 Frequently = 2 Always = 3. This scoring convention and childhood cut-off scores are taken from Cleaton et al. (2021) and Meachon et al. (2022) to adhere to more recent convention, rather than from earlier studies (Kirby et al., 2010; Rosenblum, 2013). The overall total of ≥56 is considered an indicator for being ‘at risk’ for DCD, whilst a total score of ≥65 indicates ‘probable’ DCD (pDCD). All of our DCD participants met this criterion and are thus considered to have pDCD. As discussed earlier, a score of ≥17 (Cleaton et al., 2021; Meachon et al., 2022) on the childhood section indicates the presence of coordination and movement difficulty which has interfered with daily living and education since childhood (Kirby et al., 2010). This latter point is a required DSM-IV diagnostic criterion of DCD and separates developmental DCD from those who experienced motor difficulties acquired in later life after illness or trauma.

The ADC also asks two open-ended questions; firstly, ‘Did it take you longer than others to learn to drive? (If you do not drive, please indicate why you chose not to drive)’ and ‘Can you describe any compensatory strategies or approaches that you have developed over the years in order to deal with coordination difficulties in your everyday life?’ These were not scored but some of the answers that were provided did help us understand why some adults had suspicions that they did have DCD whilst not having been formally diagnosed – for example, because their parents did not want a label attached to them, or for financial reasons. Participants also completed a visual imagery questionnaire which we do not report further here (this data is intended for a subsequent paper).

#### Diagnosing prosopagnosia

Traditionally, the PI20 has been used as a screening tool for prosopagnosia, with scores of >65 being the cut-off for identifying problematic levels of prosopagnosia symptoms (Burns et al., 2023; Shah et al., 2015). A diagnosis of DP would then be confirmed if the participant reported regular trouble with faces and scored more than two standard deviations below a control mean on the Cambridge Face Memory Test and a Famous Faces Test (Lowes et al., 2024; Pertzov et al., 2020; Tsantani et al., 2020). More recently however, it has been suggested that PI20 scores ≥60 with self-reported difficulties (in the absence of other neurological causes) should be the primary method for diagnosing prosopagnosia (Burns et al., 2023). This is because the historical method was too conservative, excluding up to 85% of people who reported severe problems in everyday life (Burns et al., 2023). We therefore used the more recent approach here to diagnose DP in our pDCD group. For completeness, however, we confirm that using the historical method for diagnosing DP (i.e. self-reported problems plus scoring >65 on the PI20, and impaired on the CFMT and FFT; Burns et al., 2023) would have resulted in 22% of our DCD group scoring within the DP range.

Participants were therefore asked if they felt they had regular trouble with faces through an initial question, ‘Do you regularly have difficulty recognising or mixing up the faces of people you should know, for example, friends, family, or famous people on TV?’. They were then assessed through the Prosopagnosia Index 20 (PI20; Shah, 2015; Table 1) to determine if their symptoms are atypically high (e.g. ≥60). These two elements are intended to form the diagnostic assessment for prosopagnosia (Burns et al., 2023). PI20 statements include: ‘My face recognition ability is worse than most people’, ‘I have always had a bad memory for faces’ and ‘I feel like I frequently offend people by not recognising who they are’. Participants reported on a five-point scale ranging from strongly disagree (1) to strongly agree (5), with five items reverse scored, for example, ‘I am better than most people at putting a face to a name’ or ‘I am very confident in my ability to recognise myself in photographs.’. Final scores can range from 20 to 100 with higher scores indicating greater face recognition difficulty. After answering these questionnaires, participants completed a variety of tests to assess their face memory and face perception abilities, respectively.

**Table 1.** Neuropsychological test results of the 32 pDCD participants.

DCD suspected or diagnosed	Age	Sex	CFMT	CFPTup	CFPTinv	Holistic perception	FFT (%)	PI20
Diagnosed	18	Female	**36**	**74**	68	−0.08	**26.1**	35
Diagnosed	26	Female	48	**68**	60	−0.12	86.2	60*
Diagnosed	28	Female	**30**	52	**84**	0.62	**48.1**	49
Diagnosed	31	Female	65	26	38	0.46	86.2	31
Diagnosed	31	Non-binary	**41**	34	**92**	1.71	86.7	80*
Diagnosed	32	Male	51	32	70	1.19	60.7	60*
Diagnosed	33	Non-binary	**28**	**92**	**102**	0.11	**37.5**	81*
Suspected	33	Female	63	28	58	1.07	77.8	30
Diagnosed	34	Female	60	40	76	0.90	93.1	59*
Diagnosed	34	Male	43	58	76	0.31	93.3	35
Diagnosed	20	Female	51	42	50	0.19	82.8	58*
Diagnosed	37	Male	**35**	**86**	80	−0.07	**4.0**	31
Diagnosed	38	Female	61	38	56	0.47	89.7	73*
Diagnosed	38	Male	**34**	**80**	80	0.00	72.4	72*
Diagnosed	48	Female	**34**	**70**	60	−0.14	**50.0**	73*
Diagnosed	51	Female	**37**	**74**	64	−0.14	**13.3**	94*
Diagnosed	56	Female	50	32	72	1.25	78.3	89*
Diagnosed	59	Female	**38**	58	78	0.34	70.0	82*
Diagnosed	73	Male	**37**	**88**	80	−0.09	**38.9**	71*
Diagnosed	20	Female	58	46	62	0.35	100.0	46
Diagnosed	21	Male	**36**	48	74	0.54	**38.5**	70*
Diagnosed	23	Female	56	**74**	64	−0.14	**44.4**	34
Diagnosed	24	Female	46	26	62	1.38	90.0	63*
Diagnosed	25	Female	**31**	**64**	78	0.22	**37.9**	75*
Diagnosed	25	Male	53	52	**86**	0.65	86.2	46
Diagnosed	32	Female	**25**	**96**	**96**	0.00	96.7	28
Suspected	24	Female	49	50	52	0.04	70.0	68*
Suspected	29	Female	59	36	64	0.78	**44.0**	53
Suspected	39	Non-binary	**40**	54	70	0.30	63.0	98*
Suspected	51	Male	52	42	64	0.52	76.7	34
Suspected	54	Female	43	**76**	**86**	0.13	**50.0**	74*
Suspected	59	Male	**38**	**68**	74	0.09	**41.4**	88*

*Note*. Cambridge Face Memory Test (CFMT), Cambridge Face Perception Test upright scores (CFPTup), Cambridge Face Perception Test inverted scores (CFPTinv) and the Cambridge Face Perception Test Holistic Perception measure (Holistic Perception), Famous Faces Test (FFT) and Prosopagnosia index questionnaire (PI20). Bold indicates impairment of two SDs using norms in the literature (CFMT: Duchaine & Nakayama, 2006; all CFPT scores: Duchaine et al., 2007), from our control sample (FFT, Holistic Perception), or from recommended cut-offs (⩾60; Burns et al., 2023) in combination with self-reported regular difficulties with faces (PI20: Burns et al., 2023).

* Indicates that the participant reported daily problems with face recognition abilities.

#### Face perception and face memory measures

The first faces task participants completed was the Cambridge Face Perception Test (Duchaine et al., 2007), which assesses whether individuals can perceive subtle differences between faces, that is, how much identity-related information can they detect. It indicates whether face-related problems are due to an inability to tell faces apart. Poor face perception suggests face-identity processing may fail at a perceptual stage prior to memory (DeGutis et al., 2014; Fox et al., 2008). The CFPT involved 16 trials that included a target face along with six test faces below the target that had been altered using computer morphing software to diverge from the target face to varying degrees, towards a separate unique identity. Participants were asked to drag the test faces into the order of ‘most like the target face’ (on the left of the screen) to the test face that looks ‘least like the target face’ (on the right of the screen) within a maximum of 60 s. Eight faces were presented upright and eight upside-down (inverted). We used norms from Duchaine et al. (2007) to identify which people with pDCD and controls were impaired on this task (Table 1).

It is thought that participants who score well on the upright trials, but poorly on the inverted trials, process faces in a more holistic way (i.e. they will perceive the whole face rather than a specific detail such as nose or lips; Burns et al., 2023; Burns & Wilcockson, 2019; Dal Lago et al., 2023, 2024), Conversely, participants who score more similarly on both upright and inverted trials are thought to rely more on specific features of a face, which is common in people who struggle to recognise faces, for example, prosopagnosia (Burns et al., 2023). By removing the inverted scores from the upright, it is thought that the remaining value indexes holistic perception. To create a holistic perception measure (Luo et al., 2017; Russell et al., 2009), we subtracted the upright errors from the inverted and then corrected it based on the participant’s upright abilities (CFPTinv – CFPTup)/CFPTup; Burns et al., 2023).

Participants then completed The Famous Faces Test (Duchaine & Nakayama, 2005). They were presented with 30 images of extremely well-known, famous people, for example, The Queen, Boris Johnson, Barack Obama and The Rock. Participants were asked to type the name of the person into a text box under the image on screen or enter other identifying details if they could not recall the name, for example ‘Harry Potter’ or ‘ex-Prime Minister’. One point was awarded for each correct answer or correctly identifying the description given. As with previous FFTs, the faces used were cropped to remove hair, background and presented in black and white so that no extraneous identity cues were visible. Participants were not given time restrictions on this task. We asked participants if they were familiar with the celebrities afterwards, so we could identify whether they had a genuine difficulty with faces, or simply did not know who they were. Percentages were computed after subtracting the number of unknown celebrities from the total possible correct. This task was used to assess long-term memory of faces that would likely be familiar to them (Duchaine & Nakayama, 2005). People with prosopagnosia often perform extremely poorly on this task compared to controls (e.g. Bate, Bennetts, Gregory, et al., 2019; Burns et al., 2023). Our famous faces test was correlated with our primary face identity processing measures (PI20: *r* = −.40, *p* < .001; CFMT: *r* = .57, *p* < .001; CFPTupright: *r* = −.46, *p* < .001; CFPTHolistic: *r* = .39, *p* < .001) thus validating it.

Finally, participants completed the Cambridge Face Memory test (Duchaine & Nakayama, 2006). This task is one of the most frequently used instruments for measuring memory for unfamiliar faces in control samples (Bate, Bennetts, Gregory, et al., 2019; Burns, et al., 2019; Childs et al., 2021; Duchaine & Nakayama, 2006; Estudillo et al., 2020; Murray & Bate, 2020) and for diagnosing DP (Adams et al., 2020; Bate et al., 2014; Bennetts et al., 2015; Biotti et al., 2016; Burns, et al., 2014; 2017ab). In each trial, participants were given 3000 ms to learn an unfamiliar face presented to them. The face was presented in three views, one facing forward, one left profile and one right profile. Participants were then shown three faces and asked to select which they thought was the target face they had just studied. One of these images was the target face, with the remaining two novel. Selecting the correct target face resulted in one point for each of the 72 trials. In the final section of the test, participants were asked to identify the targets masked with noise, making this section more challenging. Based on norms from prior work, we bold participants who exhibited substantial difficulties on this task using the −2 SD cut-off in Table 1 (Duchaine & Nakayama, 2006).

Significant differences between the pDCD and control groups were assessed using between subjects *t*-tests. To minimise the risk of a Type I error arising from the employment of multiple *t*-tests, a Bonferroni correction was calculated based on the primary measures of interest (i.e. PI20, CFMT, FFT, CFPT upright and holistic; adjusted alpha = .01). However, we do not employ the Bonferroni correction in any additional analyses that excluded DCD cases with comorbid conditions as they greatly reduce power to detect effects. These additional analyses should be considered exploratory, and confirmatory, to those of primary interest, that is, is the DCD group impaired on different face identity processing measures. We acknowledge that whilst some authors and publications advise against providing *p*-values for exploratory analyses (McIntosh, 2017), we chose to report them so meta-analyses that require exact *p*-values could utilise them (e.g. *p*-uniform; Van Assen, 2015) and *p*-curve analyses (Burns et al., 2019; Burns & Bukach, 2023; Simonsohn et al., 2014).

Ethical approval was granted by Edge Hill University Ethics Committee, and all the work was carried out in accordance with the 1964 Helsinki Declaration on human testing. All participants gave their informed consent, including for the authors to publish data derived from this work. None of our experiments or hypotheses were pre-registered. We make our data available on the Open Science Framework https://osf.io/xsp2q/ but do not provide the code to run our study as we do not own the copyright for the tasks beyond the FFT, which we do provide. When the Levene’s test for equality of variances was significant, we use a Satterthwaite approximation for the degrees of freedom as appropriate.

## Results

### Confirming probable DCD symptomology

We quantified DCD symptomology in all participants through two self-report movement questionnaires, that is, the Adolescent and Adult Coordination Questionnaire (AAC-Q; Tal-Saban et al., 2012) and the Adult Developmental Coordination Disorder/Dyspraxia Checklist (ADC; Kirby et al., 2010). To verify motor and coordination difficulties in the DCD professional diagnosed group and suspected DCD group, a one-way ANOVA was completed on the ADC and AACQ scores. Both of these were significant (AACQ: *F*[2, 66] = 106.7, *p* < .001; ADC: *F*[2, 66] = 113, *p* < .001), with the diagnosed (AACQ *M* = 44.08, *SD* = 9.34; ADC *M* = 78.68, *SD* = 19.59) and suspected (AACQ *M* = 42.71, *SD* = 12.23; ADC *M* = 82.14, *SD* = 21.64) DCD groups reporting more DCD symptoms than the controls (AACQ *M* = 17.81, *SD* = 4.05; ADC *M* = 20.65, *SD* = 12.16; all *ps* < .001). By contrast, there were no significant differences between diagnosed and suspected DCD groups (both *ps* = 1).

### DCD is associated with elevated levels of prosopagnosia symptoms

**Figure 1.**
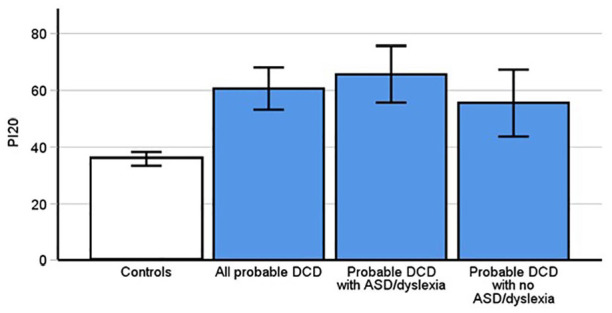
Prosopagnosia Index results. Higher scores indicate more severe symptoms of prosopagnosia. PDCD participants reported significantly higher levels of symptoms than controls (*p* < .001). This was replicated in both the subgroups of pDCD participants without ASD and dyslexia (*p* = .003), and those with pDCD and comorbid dyslexia and autism (*p* < .001). Error bars indicate 95% confidence intervals.

The PI20 questionnaire invited participants to report any facial recognition difficulties that they experience, that is, symptoms of prosopagnosia. We recently demonstrated that it is the most effective diagnostic assessment for prosopagnosia (Burns et al., 2023), with higher scores indicating greater difficulties with faces whilst low scores represent typical facial recognition ability. A between subjects *t*-test indicated that people with pDCD (Figure 1, *M* = 60.63, *SD* = 20.88] scored significantly higher on the PI20 than controls (*M* = 35.81, *SD* = 7.37, *t*[37.67] = 6.39, *p* < .001, Cohen’s *d* = 1.63). This suggests that people with DCD are more likely to report substantial difficulties with facial recognition than the general population.

We further investigated whether there was an elevated presence of comorbid developmental prosopagnosia in our pDCD cases. We therefore categorised them as having DP based on our recently recommended criteria of whether a participant reports trouble with faces in everyday life *and*, in addition, if they are reporting atypically more difficulties with faces via the PI20, that is, scores of, or above, 60. This yielded 56% of people with pDCD being diagnosed with comorbid DP (Burns et al., 2023): 5/9 males, 10/20 females and 3/3 non-binary. However, two cases that reported trouble with faces did not meet the PI20 cutoff of 60, scoring 58 and 59. Crawford’s *t*-tests confirmed significant atypicality in both cases (*ps* < .006), hinting that even the more liberal threshold of 60 may miss some self-identified prosopagnosia cases.

Highly elevated levels of prosopagnosia were replicated when we used the traditional and more conservative approach to diagnosing, that is, a score of >65 on the PI20, along with self-reported difficulties and scores poorer than −2 SDs below the control mean on the CFMT and FFT. In this instance, we diagnosed 7/32 (22%) of participants who had pDCD with prosopagnosia: 3/9 males, 3/20 females and 1/3 non-binary. In summary, our pDCD group experience comorbid prosopagnosia at a far greater rate than the estimated two percent expected in the general population (Bate & Tree, 2017; DeGutis et al., 2023; Kennerknecht et al., 2006).

### DCD may be associated with problems telling faces apart

Given that people with pDCD exhibited high levels of prosopagnosia, we were curious whether these issues were due to an inability to tell faces apart from one another. If you are unable to detect the subtle differences that make a face unique from other faces, then it is inevitable that you will have problems recognising them. To assess this, we used the upright CFPT which requires participants to detect subtle differences (and similarities) between faces. While people with pDCD exhibited elevated face perception errors, a between subjects *t*-test demonstrated that this difference was not significant (DCD *M* = 55.1, *SD* = 19.6; Control *M* = 45.89, *SD* = 21.69, *t*[66] = 1.82, *p* = .073, Cohen’s *d* = .44).

**Figure 2.**
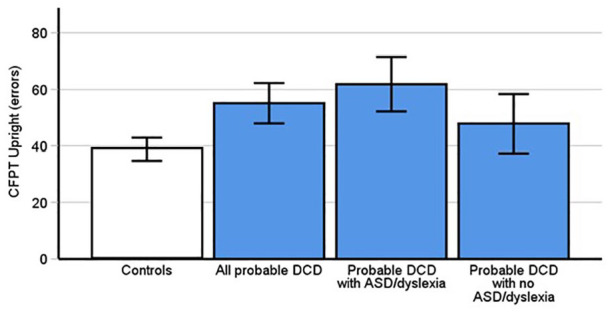
Probable DCD participants made significantly more errors on the upright Cambridge Face Perception Test (CFPT; *p* < .001), only after we added control data from Burns et al. (2023). This was replicated in a subgroup of those with pDCD and comorbid dyslexia and autism [*p* < .001], but not those without ASD and dyslexia (*p* = .09) although adding Burns et al. (2022) data did reveal problems in this subgroup (see text). Error bars indicate 95% confidence intervals.

From this result, it may appear that people with pDCD do not fail to recognise faces because they are unable to tell faces apart. However, our control group made considerably more errors on the upright CFPT than commonly used norms in the literature (Duchaine et al., 2007; Garrido et al., 2008), including our recent paper (Burns et al., 2023). We conducted further exploratory analyses by running one-sample *t*-tests using the control norms from these papers as our test statistic. This demonstrated that our pDCD group did indeed make more errors in face perception [(all *ps* < .007). We, therefore, thought it reasonable to add neurotypical data from Burns et al. (2023) to our present sample to increase power to detect a potential reduction in pDCD performance (New Control Group: *n* = 85). These exploratory analyses revealed that those with pDCD were making significantly more errors in upright face perception (Figure 2; *M* = 55.10, *SD* = 19.6) than controls (*M* = 38.8, *SD* = 18.79, *t*[114] = 4.09 *p* < .001, Cohen’s *d* = 0.86). This suggests that those with pDCD are not just self-reporting problems with faces but are, in addition, experiencing objective difficulties perceiving subtle differences between them. However, we should be cautious when interpreting these results, given they require exploratory analyses with additional data.

We had no a priori prediction for DCD being associated with difficulties on the inverted portion of the CFPT, as individuals with developmental prosopagnosia can often perform very similarly to control groups (e.g. Burns et al., 2023). However, it can serve as a test for lower-level visual perception, that is, do perceptual impairments in DCD extend to inverted faces, which are thought to rely on feature processing much more than upright faces. We therefore ran an additional *t*-test on this data to see if those with pDCD demonstrate problems on this measure. A between subjects *t*-test indicated that people with pDCD (*M* = 70.32, *SD* = 13.31) did not score significantly more errors on the inverted CFPT inverted than controls (*M* = 69.46, *SD* = 17.69), *t*[66] = 0.22, *p* = .82, Cohen’s *d* = .05). Adding data from Burns et al. (2023) did not change this (*t*[78.99] = 1.16, *p* = .25 Cohen’s *d* = .21). This suggests face identity processing difficulties in the pDCD group are restricted to upright faces only. Moreover, the lack of inverted CFPT differences arguably indicates upright face identity processing difficulties in pDCD were not likely due to attentional or low-level perception issues.

**Figure 3.**
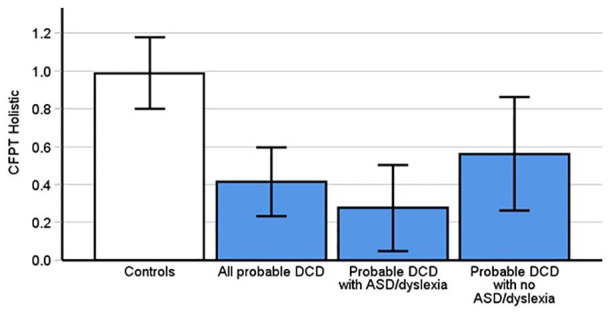
Probable DCD participants scores were significantly different than controls on the CFPT Holistic (*p* < .001) only after adding control data from Burns et al. (2023). This was replicated in the subgroup of pDCD with comorbid dyslexia or autism (*p* < .001) but not in pDCD participants without ASD and dyslexia (*p* = .07). Error bars indicate 95% confidence intervals.

The inversion effect is thought to index holistic perception (Burns & Wilcockson, 2019). Using an inversion effect computed from the CFPT scores revealed significant differences between pDCD (*M* = 0.42, *SD* = 0.5] and control participants (*M* = 0.77, *SD* = 0.8, *t*(66) = 2.16, *p* = .035, Cohen’s *d* = .53). However, it is important to note that this needs to be interpreted cautiously as it did not meet the alpha threshold after Bonferroni correction. To ensure this was not a false positive though, we again added the CFPT inversion effect data from Burns et al. (2023) to increase power. This further confirmed those with pDCD had a smaller inversion effect than controls (Figure 3, Updated Control [*M* = .99, *SD* = .87, *t*(93.02) = 4.4, *p* < .001, Cohen’s *d* = 0.72).

### DCD is associated with memory problems for unfamiliar faces

**Figure 4.**
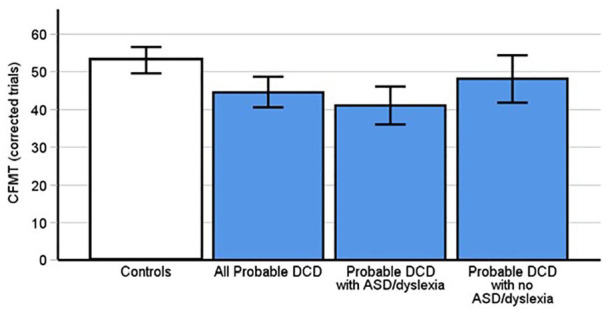
Probable DCD participants scored significantly fewer correct trials on the CFMT than controls (*p* = .002). This was replicated in the subgroup of pDCD participants with comorbid dyslexia and autism (*p* < .001), but not those without ASD and dyslexia (*p* = .14). Error bars indicate 95% confidence intervals.

The CFMT identifies participants’ abilities to recognise unfamiliar faces and has historically been used to diagnose DP (Burns et al., 2023; DeGutis et al., 2023). A between subjects *t*-test indicated that people with pDCD (*M* = 44.63 correct trials, *SD* = 11.09) scored significantly lower on the CFMT than controls (Figure 4, *M* = 53.05 correct trials, *SD* = 10.57, *t*[67] = 3.2, *p* = .002, Cohen’s *d* = .78). Thus, DCD is associated with problems remembering previously unfamiliar faces.

### DCD is associated with difficulties recognising familiar faces

**Figure 5.**
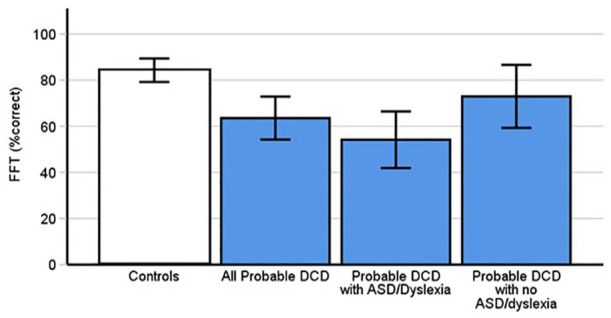
Probable DCD participants scored significantly lower on the FFT than controls (*p* < .001). Values are in percentages. This was replicated in the subgroup of those with dyslexia and autism (*p* < .001), but not pDCD participants without ASD and dyslexia (*p* = .12). Error bars indicate 95% confidence intervals.

The Famous Faces Test assesses familiar face recognition. The results of a between subjects *t*-test indicated that people with pDCD (Figure 5, *M* = 63.56, *SD* = 25.81) scored significantly lower on the FFT than controls (*M* = 84.2, *SD* = 15.18, *t*[48.57] = 3.97, *p* < .001, Cohen’s *d* = 0.99). People with pDCD therefore experience considerable difficulties in familiar face memory.

### Are difficulties with faces present in DCD after excluding autism and dyslexia?

Previous work has shown that both ASD (Cook & Biotti, 2016; Fry et al., 2022; Kracke, 1994; Minio-Paluello et al., 2020; Wilson et al., 2010) and dyslexia (Burns & Bukach, 2022; Sigurdardottir et al., 2019; Sigurdardottir et al., 2018) are linked to impaired face identity processing abilities. To avoid the possibility that our observed impairments in pDCD were attributable to these comorbid conditions, we excluded the 16 people with pDCD who reported comorbid ASD and dyslexia and ran our analyses again. We found the pDCD group (*n* = 16, 15 for CFPT measures) still reported significant difficulties in identity processing PI20 (*p* = .003), albeit not on the FFT (*p* = .12), the CFMT (*p* = .14), the CFPT upright (*p* = .76) and holistic measure (*p* = .36). Please note, we do not use Bonferroni corrections here to avoid diminishing power further given the smaller sample sizes. Further exploratory analyses without Bonferroni corrections adding Burns et al.’s (2023) data did reveal a significant difficulty on the CFMT (*p* = .004), but not the CFPT upright (*p* = .09), or the CFPT holistic (*p* = .07). Given these analyses were ran without Bonferroni corrections, the CFMT related difficulties in DCD participants without comorbidities should be interpreted with caution.

For completeness, we ran these analyses again using only the participants with pDCD who reported autism and/or dyslexia (*n* = 16). Again, we do not use any Bonferroni corrections given the small numbers. Remarkably, significant differences were found in performance between this group and controls on the PI20 (*p* < .001), the holistic measure (*p* = .024), CFMT (*p* < .001), FFT (*p* < .001) and CFPT upright (*p* = .013). Further exploratory analyses adding Burns et al. (2023) data still identified no further significant differences on the inverted perception test (*p* = .11). Thus, DCD cases with or without comorbidities still exhibited problems with faces.

### Severity of movement problems in DCD predicts face identity processing measures

We assessed whether movement difficulties were predictive of problems with faces, as previous research has shown in children (Sumner, Pratt, & Hill, 2016). Such relationships would suggest a potential overlap between face identity processing and movement-related regions of the brain, or comparatively similar levels of widespread cognitive disruption in pDCD (i.e. affecting face identity processing *and* movement areas of the brain). As the ADC contains social situation related statements which may overlap with our face-related measures, we used the AACQ in these correlations. When controls and participants with pDCD were assessed together, the AACQ was not associated with CFPT Upright (*r* = .11, 95% CI = [−0.13, 0.34], *p* = .36), CFPT Inverted (*r* = .004, 95% CI = [−0.23, 0.24], *p* = .97) or CFPT Holistic (*r* = −0.15, 95% CI = [−0.38, 0.09], *p* = .22).

**Figure 6.**
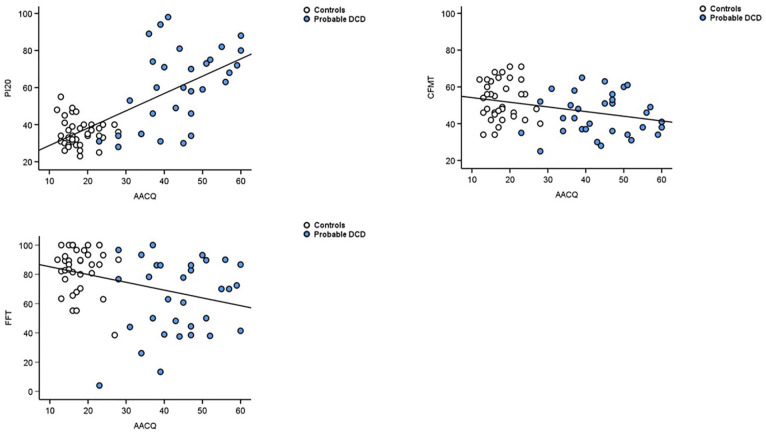
Top left panel indicates that increasing symptoms of DCD (AACQ) positively predicted prosopagnosia symptoms (PI20, *r* = .71, 95% CI = [0.57, 0.81], *p* < .001]. When analysed separately, the same correlation was not significant in the controls (white circles, *r* = −0.10, 95% CI = [−0.41, 0.23], *p* = .56), but was in DCD (blue circles, *r* = .51, 95% CI [0.19, 0.73], *p* = .003). Top right panel illustrates that increasing DCD symptomology (AACQ) negatively predicts ability to identify previously unknown faces (CFMT; *r* *=* −.33, 95% CI [−0.52, −0.1], *p* = .006). The lower left panel indicates that greater movement and coordination difficulty on the AACQ negatively predicted ability to recognise familiar faces on the FFT (*r* = −.34, 95% CI [−0.54, −0.12], *p* = .004).

AACQ scores were however correlated with the PI20 scores (Figure 6, *r* = .71, 95% CI = [0.57, 0.81], *p* < 0.001), CFMT (*r* = −0.33, 95% CI = [−0.52, −0.1], *p* = .006], and the famous faces test (*r* = −0.34, 95% CI = [−0.54, −0.12], *p* = .004]. This suggests that as movement difficulties increase, so too do prosopagnosia symptoms and face memory issues. However, when the two groups were analysed separately, only the PI20 and AACQ were significantly correlated with pDCD (*r* = 0.51, 95% CI = [0.19, 0.73], *p* = .003), again suggesting the severity of movement problems and prosopagnosia symptoms increased in tandem with DCD.

**Figure 7.**
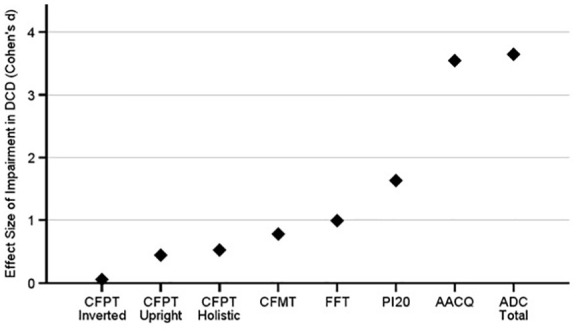
The effect sizes of impairments in pDCD across all face and movement measures. The perception related problems with faces (e.g. CFPT Upright and Holistic) were comparable to the unfamiliar face memory problems (i.e. the CFMT). Then the pDCD group’s familiar face memory (FFT) problems were midway between these and their self-reported complaints with faces in daily life (i.e. PI20). Importantly, there were minimal differences between the pDCD and control groups, as shown by the very small CFPT inverted effect size. The two movement measures are plotted for comparison purposes.

To compare the difficulties those with pDCD are experiencing at different stages of face identity processing, we plotted the effect sizes of impairments in Figure 7. We can see they are similarly medium-to-large (Cohen’s *d*: 0.44–0.78) across unfamiliar face memory (i.e. the CFMT) and perception (i.e. CFPT Upright and Holistic). By contrast, familiar face memory and self-reported prosopagnosia symptoms were large-to-very large (i.e. Cohen’s *d*: 99.–1.63). Thus, the size of the impairment detected on the famous faces test is closest to the self-reported problems in daily life as measured through the PI20. In summary, those with pDCD exhibit problems in face identity processing, including face perception and unfamiliar and familiar face memory.

## Discussion

We assessed whether there was a higher prevalence of prosopagnosia, and objective face identity processing difficulties in people with DCD. Given that facial identity processing problems are found in other neurodevelopmental conditions (Burns & Bukach, 2021; Cygan et al., 2018; Fry et al., 2022; Keogh et al., 2021; Minio-Paluello et al., 2020; Sigurdardottir et al., 2018; Svart & Starrfelt, 2022), and that such difficulties are apparent in children with DCD (Sumner, Pratt, & Hill, 2016), we hypothesised that adults with DCD would exhibit problems with face identity processing.

Our results clearly demonstrated that people with pDCD report significantly greater difficulties with faces in everyday life, as assessed through the prosopagnosia index questionnaire. These problems also manifested themselves in reduced performance on our unfamiliar and familiar face memory tests. Whilst face perception was not significantly different between our pDCD and control group on the upright and inverted tasks, our control group did score poorly compared to those tested in previous research (e.g. Burns, 2023; Duchaine et al., 2007; Garrido et al., 2008). When we performed exploratory analysis and included the control data from our recent study (Burns et al., 2023) to increase power, pDCD participants made significantly more errors in identity perception. This is important as people who struggle with the initial processing of facial identity information are subsequently going to struggle to remember them correctly. Moreover, these problems could not be accounted for by poor attention nor low level vision problems due to their intact perceptual abilities with inverted faces.

A smaller inversion effect was also found in participants with pDCD as measured through our perception test, which suggests that people with DCD potentially rely more on specific features such as eyes, nose or lips (in other words, the processing of individual features). This is in contrast to perceiving the whole face (i.e. holistic processing), which is more typical in the general population. People with prosopagnosia are often found to rely more heavily on local rather than global face identity processing strategies (Avidan et al., 2011; Liu, 2014), for example by focusing on mouths (DeGutis et al., 2012), although results supporting this hypothesis are not always consistent (Susilo et al., 2010; Tsantani et al., 2020). Our findings suggest that people with DCD may similarly rely on featural face identity processing strategies. However, it is possible that this is the result of their comorbid prosopagnosia difficulties rather than DCD itself. Nevertheless, people with pDCD appear to experience troubles extracting key information required to form coherent face identity perception. This was further reflected in the fact that our adults with pDCD showed significant problems with face recognition, often at levels that would warrant a diagnosis of prosopagnosia. Overall, people with pDCD self-report, and objectively exhibit, significantly more difficulties with face identity processing than the general population.

We also examined the prevalence of prosopagnosia in our pDCD group using recently recommended diagnostic criteria (Burns et al., 2023), that is, significantly high levels of prosopagnosia symptoms as measured through the prosopagnosia index and self-reported trouble with faces in daily life. Using this approach, we found 18/32 (56%) of pDCD individuals met the criteria to be diagnosed with developmental prosopagnosia. This is one of the largest comorbidities of prosopagnosia in the neurodevelopmental literature that we are aware of, and again highlights another comorbidity that frequently co-occurs in DCD.

We should state that the symptom-based approach to diagnosing DP has been criticised by some (DeGutis et al., 2023). While we reject these criticisms (see Burns, 2023, 2024), we do recognise that we are in a period of transition, where different authors have suggested new approaches to identifying DP (although there is a growing consensus self-reports are valid: Burns, 2023; 2024; Burns et al., 2023; Epihova & Astle, 2024; Lowes et al., 2024). Due to this, and because we used a more liberal approach to diagnosing the condition than has been used in the past (Burns et al., 2023), it is important to note that DP would still have been extremely high (22%) in pDCD even if we had adopted a more conservative approach. That is, requiring self-reported difficulties in daily life, a score of >65 on PI20, plus impairments at least 2 SDs below neurotypical norms on the CFMT and famous faces test (Burns et al., 2023). However, we note that three DCD cases met the −2 SD criteria on the CFMT and FFT but did not score atypically high on the PI20. Thus, if we had used a purely objective diagnostic approach (i.e.,impaired at the -2 SD level on only the CFMT and FFT), then 10/32 DCD cases (31%) met criteria. In any case, all three of these diagnostic approaches show that DCD and developmental prosopagnosia may be considerably overlapping, rather than distinct, conditions.

### Neurological explanations

It was not our intention to assess the extent to which those with DCD experience problems in visual domains other than faces, such as with objects or words. Prior work has suggested perception and memory problems outside of face identity processing may exist in childhood in DCD (Crawford & Dewey, 2008; Hashemi et al., 2022; Schoemaker et al., 2001; Tsai et al., 2008; Wang et al., 2017) and we believe these are likely to still be apparent in adulthood. This is because face identity and object processing impairments often co-occur in other neurodevelopmental conditions, such as dyslexia (Sigurdardottir et al., 2018; Sigurdardottir et al., 2015) and prosopagnosia (Barton et al., 2019; Burns et al., 2019; Geskin, 2020). Whilst we have shown those with DCD appear comparable to controls in terms of inverted face perception, control participants often perform more poorly on this task than the upright portion, and so it may not have been sensitive enough to detect possible domain general perceptual impairments in DCD adults. However, we have shown those with DCD demonstrate substantial problems with faces to the extent that it could be considered diagnosable as prosopagnosia. Whether this prosopagnosia is part of broader domain general visual agnosia in DCD will require further object-related testing.

By contrast, it is possible that some brain regions that deal with movement, may also process faces. For example, McLeod et al. (2014) found reduced functional connectivity between the primary motor cortex (M1) and the caudate, putamen and globus pallidus in children with DCD. This is interesting because along with controlling movement (McLeod et al., 2014), these regions may also help process visual information related to faces (Rees et al., 2012; Sugiura et al., 2000; Uono et al., 2017). It is therefore possible that disrupted connectivity between a variety of areas may affect shared motor ability and facial identity processing (Sokolowski & Levine, 2022). Biotteau (2016) also found altered functional activity in the caudate nucleus and globus pallidus during a novel motor task in children with DCD compared with controls, which further supports this notion.

Alternatively, the problems with new and familiar facial identification that have been identified in people with DCD could be explained by atypicality in the fusiform gyrus. Regions of the fusiform gyrus are implicated in both imagined and real bodily movement (Hétu et al., 2013; la Fougère et al., 2010; Michels et al., 2009; Zwergal et al., 2010) and also in face identity processing (Åsberg Johnels et al., 2022; Burns et al., 2017; Fry et al., 2022; Kanwisher et al., 1997; Zhao et al., 2022); for example, the fusiform face area (Burns & Bukach, 2023) and/or the fusiform body area, and other regions of the fusiform gyrus that process both faces and bodies (Schwarzlose et al., 2005). It may be that the facial identification issues people with DCD experience are partly due to problems in these overlapping regions of the fusiform gyrus (Schwarzlose et al., 2005), or more diffuse atypicalities that affect the broader fusiform gyrus and thus, the distinct face and body areas. Further neuro-imaging studies are required to test these hypotheses.

Other shared brain regions which may be atypical in both DCD and prosopagnosia include the cerebellum (Dinkelacker et al., 2011; Gaymard et al., 2017; Nicolson & Fawcett, 2011), dorsolateral prefrontal cortex (Dinkelacker et al., 2011; Gaymard et al., 2017) and the posterior superior temporal sulcus (de Gelder et al., 2003; Julie, 2012; Reynolds et al., 2015; Sakurai et al., 2016). These differences may also contribute to the comorbid problems we observed here. Moreover, they suggest that DCD and prosopagnosia may be overlapping conditions, with commonalities best explained by diffuse brain atypicalities (Gaymard et al., 2017).

An alternative neurological explanation suggests that under certain hormonal conditions, cortical migration caused by ectopias may occur (Ramus, 2004). Ectopias consist of 50–100 glia and neurons that escape through a breach in cortical membrane during the process of neural migration (Ramus, 2004). This causes cortical layers to become atypically arranged (known as magnocellular disruption; Ramus, 2004). Where ectopias occur in multiple regions, these cortical and white matter abnormalities (Corrow et al., 2019) may provide an explanation for underlying origins of comorbidity between neurodevelopmental conditions (Ramus, 2004). For example, if there is cortical migration in both motor regions and visual perception areas, then prosopagnosia and DCD may co-occur. Examination of potential causal explanations and pathways for neurodevelopmental differences and their comorbidity requires extensive further investigation (Corrow et al., 2019; Ramus, 2004).

### Social explanations

Several social explanations for our findings may include the social importance of age-appropriate motor movements in early development. For example, if a child does not roll over, crawl, sit, walk and run at the same age milestone as his or her peers (Farmer et al., 2017; Gaines et al., 2008; Hua et al., 2022), this may severely limit experience with faces (Sumner, Pratt, & Hill, 2016). Reduced exposure to faces may continue through development (Hua et al., 2022) as children with DCD frequently avoid team sports and group activities because such extra-curricular activities often highlight their difficulties and differences (Cairney et al., 2013; Li et al., 2018; O'Dea et al., 2020). This may then limit their social experience even more compared to their peers. Indeed, prior work has suggested that experience with faces may be an important predictor for face recognition abilities (Balas & Saville, 2015; Gandhi et al., 2017; Putzar et al., 2010; Singh et al., 2021; Walker & Tanaka, 2003). Thus, if those with DCD have less experience with faces in childhood, then this could be a potential reason for their poorer face recognition abilities in adulthood.

### Advice for clinicians

We observed such a high prevalence of prosopagnosia in our pDCD group that it is important for clinicans, researchers, parents and educators of people with DCD to be made aware of how to diagnose and support associated face recognition difficulties. First, we recommend using the prosopagnosia index (Shah et al., 2016) as the primary tool for diagnosing prosopagnosia. It is quick to administer, with scores of 60 or above, coupled with self-reported difficulties in daily life – being the simplest method for identifying that an individual has prosopagnosia (Burns, 2023; Burns et al., 2023). A recent version has also been created for children suspected of DP using parental self-report (Lowes et al., 2024). If cases are reporting troubles, but fail to meet criteria on the PI20, then they could be assessed using objective tests, like the CFMT and FFT, as employed here. Once a diagnosis has occurred, appropriate advice can be given to the individuals concerned as to how cope more effectively in everyday life, including some quite simple measures like seeking assistance from others to help them name people they are talking to (Adams et al., 2020), and requesting additional preparation time prior to meetings (Adams et al., 2020). There are also computer-based training paradigms that may be of assistance to some people with prosopagnosia (Bate et al., 2015; Bate et al., 2022; Behrmann et al., 2005; DeGutis et al., 2014; Liu & Behrmann, 2014). Future work will be required to assess how effective these are for people with DCD.

Some authors have highlighted that DCD can be associated with considerable social difficulties (Mandich et al., 2003; Tal-Saban & Kirby, 2020; Tal Saban & Kirby, 2019; Zwicker & Lee, 2021). Indeed, it has been suggested that social problems may be a key feature of DCD, and thus researchers have included items related to this in the ADC symptom questionnaire (Kirby et al., 2010). However, prosopagnosia poses obvious difficulties in social interactions and may be associated with elevated social anxiety (Corrow et al., 2016; Davis et al., 2011) and interpersonal problems more generally (Dalrymple et al., 2014; Yardley et al., 2008). Our findings therefore suggest comobid prosopagnosia could at least partially explain why these problems are also experienced by those with DCD.

### Limitations

We note that despite reports that DCD is more prevalent in males (Ke et al., 2023; Missiuna & Polatajko, 1995), only 37.5% of our pDCD group were men. However, greater female participation in DCD studies is common (Job et al., 2019; Kirby & Saban, 2019; Scott-Roberts & Purcell, 2018; Tal-Saban et al., 2012). This is perhaps because females are more likely to respond to calls to participate in research generally (Lobato et al., 2014; Singer et al., 2000). Alternatively, and speculatively, perhaps DCD is more prevalent in females and non-binary individuals than previously thought. Gender issues have been raised in the diagnosis of ASD and ADHD (Lai et al., 2015; Mowlem et al., 2019; Rynkiewicz et al., 2016; Warrier et al., 2020), and this may also be worthy of consideration in future DCD research.

In this study, we were limited to online data collection due to COVID-19.

This prevented any in-person movement testing of participants. However, as discussed earlier, no validated adult movement battery is currently available (Meachon et al., 2022; Purcell et al., 2015). We could have asked participants to forward diagnostic documentation; however, this was not allowed due to our ethical approval (Meachon et al., 2022). Plus, it would have placed an unnecessary burden on participants with DCD, and so we based our methods upon best practice from previous studies using validated measures of behavioural difficulties (Cleaton et al., 2021; Engel-Yeger, 2020; Engel-Yeger & Engel, 2023; Harris et al., 2021; Meachon et al., 2022; Tal-Saban et al., 2012). Such validated self-report measures combined with participants’ reports of clinical diagnoses are often accepted in neurodevelopmental condition research, (e.g. Chandler, 2018; Karlsson et al., 2013; Protopapa & Smith-Spark, 2022; Abu-Akel et al., 2019; Lopes et al., 2020).

We did not include tests of visual perception in other domains such as objects or words. After pilot testing, it was established that the time and cognitive burden upon participants would have been too great if we were to include such additional tasks. However, the largely comparable scores between control and pDCD groups on the inverted CFPT showed that our results were unlikely to be due to problems with lower-level visual perception. It is possible the task may not have sufficient sensitivity to detect higher level domain general impairments in DCD that may be apparent for non-face stimuli like cars or words. We recommend future testing to assess whether perception and memory difficulties extend beyond faces in people with DCD. Moreover, it is important to note that regardless of the whether comorbid memory problems are apparent with objects, that does not alter the fact that the difficulties many with pDCD experience with faces is at the level we expect in prosopagnosia. It has been argued recently that such DCD cases should be considered part of the heterogeneous prosopagnosia population, regardless of co-occurring problems with broader object recognition (Epihova & Astle, 2024), and that is a position we endorse. However, the presence of similarly substantial problems with object and object expertise memory in DCD (Barton et al., 2019; Burns et al., 2019; Burns & Bukach, 2023; Gauthier et al., 1999) would support a more global, neurocognitive disruption account of DCD.

Additionally, some caution should be taken in accepting the lack of influence other comorbid neurodevelopmental conditions may have on face identity processing in DCD. For example, we found ASD and dyslexia may have played a role in face identity processing difficulties in pDCD, that is, when we excluded participants with comorbid ASD and dyslexia, we could only confirm self-reported and unfamiliar face memory troubles. This suggests that ASD and dyslexia may contribute to some of the group difficulties with face perception and familiar face memory, rather than DCD itself. Although it is important to note that the direction of mean performance in the DCD group without comorbidities was still in the expected direction (i.e. impaired), so the lack of effects may have been due to low power. Additionally, these diagnoses were based on participants’ self-reported diagnostic status, we did not formally assess the presence of these conditions ourselves (e.g. using an autism symptom questionnaire like the Autism Quotient; Baron-Cohen, 2001). It is possible that some in the DCD comorbidities absent group may have had these conditions, albeit undiagnosed or unsuspected. Similarly, depression is often associated with problems in memory, and occurs at higher rates in neurodevelopmental conditions (Eyre et al., 2019; Hollocks et al., 2019; Hudson et al., 2019; Oddo et al., 2016; Riglin et al., 2021), including DCD (Åsberg Johnels et al., 2022; Berggren et al., 2016; Brady et al., 2020; Cairney et al., 2013; Engel-Yeger & Engel, 2023). While most face recognition studies in neurodevelopmental conditions do not assess the role of depression (Fry, 2022; Hadjikhani et al., 2004; Hadjikhani et al., 2007; Lazar et al., 2014; Omer et al., 2019; Riby et al., 2008; Sigurdardottir et al., 2018; Sigurdardottir et al., 2015; Walsh et al., 2016; Webb et al., 2017; Weigelt et al., 2012; Whyte et al., 2016), we are unable to rule out its potential influence in our study. Future work could remedy these issues by formally assessing the role such conditions may play in face identity processing abilities in DCD.

We have previously shown that when self-identified prosopagnosia cases score atypically high in their PI20 symptoms, they will exhibit objective impairments in face identity processing on multiple tasks (Burns, 2024; Burns et al., 2023). Moreover, across 61 self-identified prosopagnosia cases and three controls who reported suspected prosopagnosia, we did not find any that failed to exhibit atypical symptoms levels on the PI20 (Burns, 2024; Burns et al., 2023). By contrast, in our Results section, we found three of our DCD cases performed atypically poorly at the -2 SD level on the FFT and CFMT, but failed to score atypically on the PI20. Importantly, and in agreement with these cases’ normal PI20 scores, these individuals did not self-identify as having developmental prosopagnosia, and so we did not diagnose them as such. However, given their severe difficulties on multiple objective tests, it is possible they do have a form of developmental prosopagnosia that is not observable from their PI20 scores. Currently, there are only two papers that have explicitly assessed the symptom-based approach to diagnosing prosopagnosia (Burns et al., 2023; Burns, 2024), and we anticipate there will be some refinement to this method as new data is collected. The PI20 has never been edited as a diagnostic tool of prosopagnosia by a group independent of the original developers, despite this being recommended in scale development (Boateng et al., 2018). Some adjustments in the PI20 may therefore be required to improve its sensitivity for detecting abnormality in prosopagnosia cases like the three we have identified here.

## Conclusions

The prevalence of prosopagnosia in people with pDCD is considerable – estimated at 56% using a symptom approach (Burns, 2023); 31% using a cognitive task-based approach; (Djouab et al., 2020; Fisher et al., 2020; Stumps et al., 2020) and 22% using conservative self-report and task-based criteria (Burns et al., 2023). We have shown that DCD difficulties in adulthood are not just restricted to movement but can often include serious problems when recognising faces. We suggest that clinicians, educators, parents and people who have DCD should be made aware of this. The PI20 is a simple test to undertake, and with an appropriate diagnosis, relevant support could be put in place. Future research must aim to clarify the nature of the possible link between these conditions, as it could lead to a major reconceptualisation of the two being related and overlapping, rather than as separated and distinct, neurodevelopmental conditions. This would be a major step forward in our understanding of both DCD and DP.

## Supplemental Material

sj-docx-1-qjp-10.1177_17470218241275977 – Supplemental material for Prosopagnosia is highly comorbid in individuals with probable developmental coordination disorder Supplemental material, sj-docx-1-qjp-10.1177_17470218241275977 for Prosopagnosia is highly comorbid in individuals with probable developmental coordination disorder by Katherine Jane Maw, Geoffrey Beattie and Edwin Burns in Quarterly Journal of Experimental Psychology

## Notes

1. The 20-year-old female scored below cut-off on the FFT, but not the CFMT. Of the two control cases reporting trouble with faces, one scored below cut off on the CFMT, but not the FFT. The other scored in the normal range on the CFMT and FFT.
