# Supplementary material for: Prosopagnosia is highly comorbid in individuals with probable developmental coordination disorder
Source: Q J Exp Psychol (Hove). 2024 Aug 13;78(8):1501–22. doi: 10.1177/17470218241275977 (PMC12267868; doi:10.1177/17470218241275977)
Supplement: sj-docx-1-qjp-10.1177_17470218241275977 – Supplemental material for Prosopagnosia is highly comorbid in individuals with probable developmental coordination disorder [file sj-docx-1-qjp-10.1177_17470218241275977.docx]

**Supplementary Information**

**Inclusion Criteria.**

**Prosopagnosia Excluded Participants Comparison.**

In the study, we included control participants who disclosed having some troubles with faces in daily life as this is representative of the general population. To ensure inclusion of these two people did not affect the results we presented, we re-ran analyses with these two participants excluded.

Results of between subjects *t*-tests continued to demonstrate significant differences between pDCD [*M*= 60.63, *SD*= 20.88] and control groups [*M*= 35.49, *SD*= 7.33, *t*(37.95) = 6.46 *p* <.001, *Cohen’s d* = 1.64] on the PI20 self-report questionnaire when the 2 participants were excluded.

Our findings on the CFPT upright also demonstrated significantly higher scores in the pDCD group [*M*=55.1, *SD*= 19.6] than control groups [*M*= 38.63, *SD*= 18.89, *t*(112) = 4.1 *p* < .001, *Cohen’s d* = .86] when power was increased by including data from Burns (Burns et al., 2022). Excluding participants declaring problems with faces did not alter the significance of these results, or the non-significant results on the CFPT inverted task.

Having excluded the two participants who reported troubles with faces in daily life, the statistically significant effects that we observed originally on the FFT [*p* < .001] and CFMT [*p* = .001] remained. They also remained significant for the CFPT holistic [*p* <.001] measures when Burns et al. (2022) were added.

**Probable DCD Excluded Participants comparison.**

In the study we excluded pDCD participants who did not meet the cut off criteria for movement and coordination problems in childhood. As DCD is, by definition in the DSM-5 criteria, a condition present since early childhood, we excluded the two participants who did not meet this criteria from our pDCD group. However, to ensure this did not alter our results greatly, we have also conducted between subject *t-*tests on face recognition tasks with these two participants included.

Results demonstrated that the significant differences between pDCD [*M*= 59.32, *SD*= 21.21] and control groups remained [*M*= 35.81, *SD*= 7.37, *t*(40.28) = 6.13 *p* < .001, *Cohen’s d* = 1.51] on the PI20. Adding the additional two did not alter the significant results on the CFMT [*p* = .002] or the FFT test [*p* <.001].

The significant differences between pDCD [*M*= 54.73, *SD*= 19.17] and control groups remained [*M*= 38.80 *SD*= 18.79, *t*(116) = 4.11 *p* <.001, *Cohen’s d* = .84] on the CFPT upright and CFPT holistic task [*p* < .001] with Burns et al. (2022) control data added. There also remained no differences between the groups on the CFPT inverted [*p* = .2].
